# My Path from Chemistry to Phytochrome and Circadian Rhythms

**DOI:** 10.3389/fpls.2016.00261

**Published:** 2016-03-15

**Authors:** Elaine M. Tobin

**Affiliations:** Department of Molecular, Cell and Developmental Biology, University of California, Los AngelesLos Angeles, CA, USA

**Keywords:** phytochrome, circadian rhythms, CCA1, photoperiodic flowering, LHY

## Abstract

I summarize my scientific journey from my first interest in science to my career investigating how plants use the phytochrome photoreceptor to regulate what genes they express. I then describe how this work led to an understanding of how circadian rhythms function in plants and to the discovery of CCA1, a component of the plant central oscillator.

As a child growing up in Louisville, Kentucky, I was interested in science classes and scientific things, but this was in the 1950's and in a place where women didn't generally have a career or any opportunities for a scientific career other than teaching. In 8th grade I did a massive report on “atomic energy.” In high school I especially enjoyed my chemistry and biology classes. I particularly enjoyed dissecting animals. One still did that in a biology course, as well as making leaf and insect collections. I even did a little outside the class investigations, including dissecting a dead owl that an aunt had found and discovering that it had a crop full of wasps, which, I concluded, probably led to its death. As a senior, I was able to take a “chemistry 2” course which had only five students and my teacher didn't seem to mind having a girl in the class. When I graduated in 1962 he gave me a gift of a paper-back book about chemistry with the inscription “to a fine chemist, and a girl at that.” My family doctor had a female medical technician who did his lab work on site, so that was the first possible opportunity that I saw for myself. He also introduced me to a scientist at the medical school who let me hang out in his laboratory one summer, though not really as a participant.

I was a chemistry major at Oberlin College and didn't take a biology course until my junior year. It was in one of those courses that I learned (from Tom Scott, one of Winslow Briggs' early students) about phytochrome, which had been discovered relatively recently. I was fascinated by it, particularly by the fact that light could cause a chemical change in a molecule that led to the regulation of biological processes. Discouraged by Professor Scott from applying to medical school (“If you want to do research, medical school will ruin you…you need to go to graduate school”), I thought graduate work in biochemistry would be appropriate. I was accepted by the Stanford biological sciences department. After graduating in 1966, off I went to Palo Alto, where I took a wonderful biochemistry course given by the medical school biochemists and then discovered that the department would not accept women as Ph.D. students. In the meantime, I also took Professor Briggs' course in what was then called plant physiology and rotated in his laboratory. Wow! He evidentially wasn't aware that women couldn't be good scientists, so he treated me like I could be and let me do experiments that I thought would be interesting. When he moved to Harvard the next year, I was happy to transfer there so I could work with him for my thesis.

It was in Winslow's classes that I learned about phytochrome and photoperiodic flowering and the work that Karl Hamner had done on the influence of circadian rhythms on flowering. Hamner had used soybeans, a short day species, to demonstrate that it was the length of the dark period, rather than the length of the light period that was the important factor for its photoperiodic flowering (Hamner, 1940). Further, by using extended dark periods of varying lengths, he showed that there was a circadian rhythm of effectiveness of the dark period (Hamner, 1960). Then, Cumming et al. (1965) showed that during an extended dark period (72 h, which gave an intermediate flowering response) a brief red interruption at various times showed a circadian pattern of response that could either increase or decrease flowering, depending on the time from the beginning of the dark period that it was given. Thus, the connection between phytochrome and circadian rhythms was brought to my attention early in my graduate career.

For my thesis work, I studied physical properties of partially purified phytochrome (Tobin and Briggs, 1973). In this work I concluded “…The difference spectra (comparing P_r_ and P_fr_) may reflect changes in chromophore absorbance and in the environment of amino acid residues near the chromophore, particularly of tyrosine, and perhaps of tryptophan and cysteine.” Now, of course, we know in great detail what happens, thanks to Clark Lagarias and Rick Vierstra and their colleagues. Winslow was a wonderfully supportive advisor. He found me a fellowship to spend a year at the Weizmann Institute in Israel where my new husband was doing his post-doc. After I returned, he allowed me to bring David, my first baby, into the lab with me until he was old enough for the day care center adjacent to the Bio Labs. I think it helped that he also had a new daughter at about the same time.

For my post-doctoral studies I worked in the laboratory of Attila Klein at Brandeis. At this time, new methods were being developed that I enthusiastically took up. Bryan Roberts had just developed an *in vitro* system for translating mRNA by isolating polyA RNA (Aviv and Leder, 1972) and incubating it with a wheat germ extract that allowed for its translation in the presence of a radiolabeled amino acid (Roberts and Paterson, 1973). I was lucky to have met with Bryan Roberts during a visit to Israel and have him show me his method for making the extract. The radioactive translation products could then be visualized by subjecting them to electrophoresis on a polyacrylamide gel and examining the radioactivity in the gel. The first such experiments I did were done with “tube gels,” which after electrophoresis were freed from the glass cylinder, frozen, sliced with a microtome and then put into tubes sequentially to measure their radioactivity. One afternoon in the library (one still looked at the latest journals there every week) I came across a paper by Bonner and Laskey (1974) describing the use of slab gels. Realizing what an improvement that would be, I rushed to the workshop at Brandeis and had them make me such an apparatus. Drying down the gel before exposing it to film proved slightly dangerous in my hands—I was rather badly scorched on one hand, just before an appointment with my obstetrician. He looked at it, turned slightly pale, and asked me to cover it up while he did his examination. He later successfully delivered my second son, Adam, who as a baby accompanied me to my work at Brandeis. Adam was sometimes looked after by Martin Gibbs, the longtime editor of Plant Physiology, who had his office adjacent to the Klein lab, while I worked in the lab. My work there resulted in a publication demonstrating that the mRNA isolated from plants given a 24 h dark treatment and then returned to light for 24 h resulted in differences in the mRNA products—thus showing that a light treatment altered what mRNAs were made or, to be more precise, what mRNAs accumulated (Tobin and Klein, 1975).

Next, thanks to an affirmative action appointment for me and a job offer to my husband, I got a job in the Biology Department at U.C.L.A. When I arrived in the summer of 1975 the department wasn't enthusiastic about providing me with either lab space or research money. They initially let me use a desk that belonged to a graduate student doing field research and it was there that I wrote my first grant application. In the meantime, I was told I could use a bench in a lab space that I had thought was intended for me, but that was disputed by a senior professor who thought it was his space. However, I was blessed to be supported by another senior professor, the previously mentioned Karl Hamner, who was winding down his research before retiring and actually let me use some of his left over grant money for supplies and to hire a technician! And then my own grant was funded…still I needed a lab of my own. I went to the head of the department “space committee” for help. He said just find some space for yourself and we will approve it. So I “found” the Hamner space, which was in the windowless basement of a building that had not been well maintained, but I was happy to have my own space. Furthermore, it seems that Hamner's studies of circadian rhythms permeated the building and thus ultimately helped lead my research in that direction.

My initial work at U.C.L.A. was carried out with undergraduate volunteers, a technician and myself. We made antibodies to both the small subunit of Rubisco (thanks to help with isolating it from Sam Wildman's lab) and a chlorophyll a/b-binding protein (thanks to help from Philip Thornber's lab). We were then able to use these to demonstrate that the mRNAs for both these proteins increased in response to phytochrome action (Tobin, 1981; Stiekema et al., 1983; Karlin-Neumann et al., 1988). Still, by just looking at mRNAs one couldn't know whether an increase was the result of new transcription or decreased degradation. My English post-doc, Jane Silverthorne, was able to demonstrate that it was indeed an increase in transcription, using nuclei isolated in the dark from dark-grown plants that had been treated (or not) with red and/or far red light (Silverthorne and Tobin, 1984). This was the first proof that phytochrome action could affect transcription of specific genes. Soon after we wrote a review of light-regulated gene expression in plants (Tobin and Silverthorne, 1985) that later became a “citation classic” (Tobin and Silverthorne, 1993).

We then started experiments to try to understand what it was about these phytochrome regulated genes that enabled them to respond to phytochrome action. We examined one of the genes for the chlorophyll a/b binding proteins (Lhcb genes, also known as cab genes) and identified a region of its promoter that was necessary for its phytochrome responsiveness (Kehoe et al., 1994; Kenigsbuch and Tobin, 1995). We then were able to isolate a clone for a transcription factor that bound to this region, which was rich in cytosines (C) and adenines (A), and we called it CCA-1 (Wang et al., 1997). It wasn't long after that Zhi-Yong Wang expressed the CCA1 clone under a constitutive CaMV 35S promoter in Arabidopsis plants. He noticed that when he watered the plants their leaf positions varied, depending on whether he was watering them in the morning or at night. Chance favors the prepared mind, and mine had been prepared by Winslow's class long ago…. The circadian rhythms were affected! We went on to show that every rhythm we observed was gone and that the endogenous CCA1 gene was repressed. Thus, CCA1 must almost certainly be a component of the central oscillator in plants. Zhi-Yong went to a meeting with these results on a poster and at the meeting someone had a look at it and said, “That looks very much like what I have seen from George Coupland's lab in England.” Panic! However Coupland had a different, but closely related gene (LHY) and he was cooperative so we were able to submit our work together. The papers were published in the same issue of Cell, though I had to argue vociferously over the phone with Ben Lewin, then the editor, that these papers were a real and important breakthrough in identifying two components of the central oscillator in plants (Schaffer et al., 1998; Wang and Tobin, 1998).

Both CCA1 and LHY fulfill important criteria for a central oscillator component. They are expressed with a circadian rhythm, constitutive expression stops rhythms, they repress their own expression, and a final criterion, that an abrupt transient increase (a pulse) in their expression resets the clock. The last of these has seldom been tested in the model systems used to study circadian rhythms. It had been tested for KaiC in cyanobacteria (Ishiura et al., 1998), for FREQUENCY in Neurospora (Aronson et al., 1994) and for PERIOD in Drosophila (Edery et al., 1994). My student, Steve Knowles, set out to test it for several proposed components in the plant clock. In order to give a pulse of each component, he utilized the ethanol-inducible system from *Aspergillus nidulans* (Caddick et al., 1998; Roslan et al., 2001) to make it possible to induce the proposed component at any time during the circadian cycle by a brief exposure to ethanol. He found that an ethanol induced pulse of either CCA or LHY could indeed reset the phase of circadian rhythms of gene expression (Knowles et al., 2008). Thus, while it has long been widely accepted, this work definitively proved that CCA1 and LHY are part of the central oscillator in plants.

## Author contributions

ET contributed to all the work described in this paper and supervised the members of her laboratory who carried out the experiments along with her.

## Funding

The research discussed here was supported by a grant from the National Institutes of Health, GM23167 and the Office of Extramural Research.

### Conflict of interest statement

The author declares that the research was conducted in the absence of any commercial or financial relationships that could be construed as a potential conflict of interest.
